# Does left atrial volume affect exercise capacity of heart transplant recipients?

**DOI:** 10.1186/1749-8090-5-113

**Published:** 2010-11-17

**Authors:** Mohammad Abdul-Waheed, Mian Yousuf, Stephanie J Kelly, Ross Arena, Jun Ying, Tehmina Naz, Stephanie H Dunlap, Yukitaka Shizukuda

**Affiliations:** 1 Division of Cardiovascular Diseases, Department of Internal Medicine University of Cincinnati, Cincinnati, Ohio, USA; 2 UC Health, Cincinnati Ohio, USA; 3 Department of Physiology and Physical Therapy, Virginia Commonwealth University, Richmond, Virginia, USA; 4 Department of Internal Medicine, Virginia Commonwealth University, Richmond, Virginia, USA; 5 Department of Public Health Sciences, University of Cincinnati, Cincinnati, Ohio, USA; 6 Cincinnati Veterans Affairs Medical Center, Cincinnati, Ohio, USA

## Abstract

**Background:**

Heart transplant (HT) recipients demonstrate limited exercise capacity compared to normal patients, very likely for multiple reasons. In this study we hypothesized that left atrial volume (LAV), which is known to predict exercise capacity in patients with various cardiac pathologies including heart failure and hypertrophic cardiomyopathy is associated with limited exercise capacity of HT recipients.

**Methods:**

We analyzed 50 patients [age 57 ±2 (SEM), 12 females] who had a post-HT echocardiography and cardiopulmonary exercise test (CPX) within 9 weeks time at clinic follow up. The change in LAV (ΔLAV) was also computed as the difference in LAV from the preceding one-year to the study echocardiogram. Correlations among the measured parameters were assessed with a Pearson's correlation analysis.

**Results:**

LAV (n = 50) and ΔLAV (n = 40) indexed to body surface area were 40.6 ± 11.5 ml·m^-2 ^and 1.9 ± 8.5 ml·m^-2·^year^-1^, data are mean ± SD, respectively. Indexed LAV and ΔLAV were both significantly correlated with the ventilatory efficiency, assessed by the VE/VCO_2 _slope (r = 0.300, p = 0.038; r = 0.484, p = 0.002, respectively). LAV showed a significant correlation with peak oxygen consumption (r = -0.328, p = 0.020).

**Conclusions:**

Although our study is limited by a retrospective study design and relatively small number of patients, our findings suggest that enlarged LAV and increasing change in LAV is associated with the diminished exercise capacity in HT recipients and warrants further investigation to better elucidate this relationship.

## Introduction

The exercise capacity of heart transplant (HT) recipients is reportedly 30 to 40% lower than age/sex matched apparently healthy individuals [1-4]. Mechanisms for this limitation are suggested to be multifactorial. Denervation, altered response to catecholamines, tissue damage due to rejection episodes, general deconditioning associated with heart failure prior to HT, and long-term use of immunosuppressant drugs have all been proposed, but conclusive data for each mechanism is lacking [2]. Renlund et al. have reported that although longer donor heart ischemic time and frequent rejection have no effect, elevated resting pulmonary vascular resistance inhibits exercise capacity [2]. Similarly, animal models of heart denervation both with chemicals [5,6] and HT [7] show no indication of a decrease in cardiac function during exercise due to denervation. Therefore, the factors, which limit exercise capacity of HT recipients, remain undefined.

Recently, increased left atrial volume (LAV) has been reported to predict diminished exercise capacity in patients with heart failure [8] and hypertrophic non-obstructive cardiomyopathy [9]. One proposed mechanism is that expanded LAV could be a reflection of chronic left ventricular (LV) diastolic dysfunction, either at rest or during exercise, which may in turn impair exercise capacity [8,9]. Another possible aspect of altered left atrial function [10,11] in HT recipients is that suboptimal active contraction in a presence of dilated left atrium and the surgical scar of the anastomosis between native and donor atrium in post-transplant may diminish left ventricle preload and thus further limit exercise capacity caused by LA enlargement itself. Therefore, we hypothesized that increased LAV is associated with diminished exercise capacity in HT recipients, and used echocardiography and cardiopulmonary exercise testing (CPX) to evaluate their relationship.

## Design and Methods

### Study population

**Figure 1:**
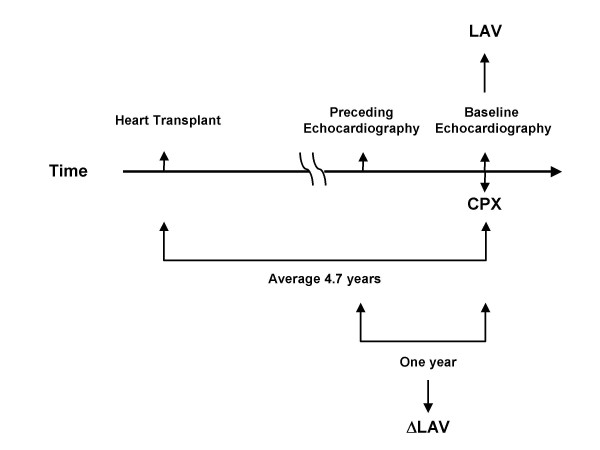
**Study design**. The study design is shown. Left atrial volume (LAV) was calculated from baseline echocardiography and the volume change in LAV (ΔLAV) was calculated from the baseline LAV subtracted that at the preceding one year. CPX = cardiopulmonary stress test.

This clinical protocol was approved by the Institutional Review Board and was consistent with the principles of the Declaration of Helsinki [12]. Due to the retrospective nature of the study, waiver of consent was approved. Patients with heart failure who underwent post HT clinical follow up were included when the following conditions were met: 1) Post HT follow up was performed in our institution, 2) Baseline post-HT echocardiography was performed within 9 weeks of post transplant CPX, 3) No more than mild mitral regurgitation during baseline echocardiograph, 4) No clinically significant myocardial ischemia with stress testing at the time of study entry, 5) Normal sinus rhythm, 6) No clinically significant active transplant rejection at the time of study entry, and 7) No prescription of β-adrenergic receptor blocker at the time of CPX. The study design for the present investigation is illustrated in Figure 1. Fifty out of a potential 108 patients who visited our clinic for a post HT follow up between 1998 and 2007 met the inclusion criteria. Among them, 48 patients received HT at our institution and 2 patients received HT at an outside hospital. Among the patients studied, 45 patients received standard right atrial anastomosis and 3 received bicaval anastomosis. The type of right sided anastomosis could not be determined in two cases. All cases received standard left atrial cuff anastomosis. In 40 cases, echocardiography at one year prior to the baseline echocardiogram was available to calculate the change in the LAV. By the study design, CPX was not performed to evaluate a change in exercise capacity during this one year interval to calculate the change in the LAV. The time duration after HT to the echocardiography conjunction for the CPX analysis was within 2 years in 11 patients, between 2 years and 5 years in 18 patients, and more than 5 years for the remaining patients.

### Echocardiographic measurements

The patients were imaged with multifrequency transducers with center frequencies of 2.5 or 3.5 MHz (ATL HDL 1000, Philips Medical system, Bothell, Washington, USA, iE33, Philips Medical System, Bothell, Washington, USA, Vivid 7 GE Healthcare system, Milwaukee, Wisconsin, USA). Briefly, in all cases pulmonary veins and the LA appendage were excluded from planimetric analysis. The outline of the atrial endocardium was traced at the end of ventricular systole at the point of maximum LA dimension. Studies were recorded digitally and stored in the Camtronics Imaging system (Emageon Camtronics system, Birmingham, Alabama, USA). Left atrial volume measurements were performed off-line on digital loops using a Digisonics review station (version 3.2 software, Digisonics Inc. Houston, Texas, USA) as previously reported by our group [9,13,14]. LAV were measured using the hand four chamber views at end systole [9,13,14]. We used this method over the area-length method recommended by the American Society of Echocardiography [15] to calculate LAV because our method is based by fewer geometric assumptions than the area-length method. In our preliminary study, the interobserver variability of non-indexed LAV was 13.5 ± 2.0% volume, n = 19 and intraobserver variability was 8.8 ± 1.5% volume, n = 23 (values are mean ± SEM). These findings were typical noted for volumetric measurements based on 2-dimensional echocardiography [15]. The one-year change in LAV (ΔLAV) was computed as a difference between left atrial volume measurements in the same patient one year apart. Additionally, left ventricular volume and ejection fraction were calculated from apical 4 and 2 chamber views using the biplane Simpson method [15]. Left ventricular diastolic function was assessed in all patients using pulsed Doppler peak E, A velocities, and E/A of mitral inflow as previously described [16]. The tissue Doppler imaging of lateral mitral annulus was also performed to measure peak diastolic E' velocity and E/E' ratio was calculated to assess left ventricular diastolic function as previously described [17]. The studies were blinded and measured by a single reader (Y.S.).

### Cardiopulmonary Exercise Testing

Exercise tests were performed on a treadmill using a ramping protocol, which is appropriate for patients with a diminished aerobic capacity [18-20]. Briefly, the starting speed and grade were 27 m·min^-1^and 0% respectively. After 2 min of exercise the speed plateaued at 64 m·min^-1 ^then the grade was increased by 0.5% every 15 seconds. Throughout the test, ECG, symptoms, blood pressure, and respiratory gas analysis were recorded. Ventilatory expired gas analysis was performed by a metabolic cart (Medgraphics Ultima, Medgraphics, St. Paul, Minnesota, USA) [21,22]. The oxygen and carbon dioxide sensors were calibrated prior to each test using gases with known oxygen, nitrogen, and carbon dioxide concentrations. Test termination criteria consisted followed American Heart Association/American College of Cardiology guidelines [23]. Oxygen consumption, VO_2 _(ml·kg^-1·^min^-1^), Carbon dioxide production, VCO_2 _(L·min^-1^), and minute ventilation, VE (L·min^-1^) were collected throughout the exercise test. Peak VO_2 _was expressed as the highest 30-second average value obtained during the last stage of the exercise test. Peak respiratory exchange ratio (RER) was the highest 30-second averaged value during the last stage of the exercise test. Ventilatory efficiency was assessed by the VE/VCO_2 _slope as previously reported with higher values (steeper VE to VCO_2 _relationship, normal < 30) reflect limited exercise capacity and abnormal cardiopulmonary physiology [9,13,24].

### Statistical Analysis

Data are presented mean ± SD. for measurements. The relationship between both LAV and ΔLAV and CPX variables were analyzed by a Pearson correlation test. The correlation between CPX variables and time since HT was also assessed. Exercise parameters between the patients with positive and negative values of indexed ΔLAV were compared with an unpaired Student t-test. All tests were two-sided and analyses with a p-value < 0.05 were considered statistically significant.

## Results

### Patients' characteristics

Among the patients investigated, most were asymptomatic [36 patients (72%) were NYHA class I] and although 48% of the patients had a history of histological-determined transplant tissue rejection in the past, all were subclinical with less than International Society for Heart and Lung Transplantation grade II (Table 1). The etiology of heart failure resulted in HT was non ischemic in 22 patients, ischemic in 27 patients, and combined non ischemic and ischemic in 1 patient. Baseline echocardiography showed that the patients had normal left ventricular systolic and diastolic function demonstrated by normal peak E tissue velocity of the mitral annulus (Table 2). The estimation of left atrial pressure, E/E' [17,25], was also within the normal range for this group. The average of left atrial volume indexed to body surface areas was significantly larger than normative values (indexed left atrial volume < 34 ml·m^-2^) [9], reflecting typical HT morphology and 32 patients (64%) demonstrated indexed atrial volume > 34 ml·m^-2^. The indexed ΔLAV was 1.9 ± 8.5 ml·m^-2·^year^-1^, indicating a relatively small increase in the LAV over the one year observation period in this cohort. In our population, the average baseline systolic blood pressure was 125 ± 18 mmHg and the baseline diastolic blood pressure was 78 ± 11 mmHg. Only 4 subjects demonstrated clinically significant hypertension (systolic blood pressure > 150 mmHg or diastolic blood pressure > 95 mmHg). In addition, no significant correlation was noted between baseline blood pressures and parameters of exercise capacity.

**Table 1:** Baseline Characteristics

Variables	N = 50
Age	57 ± 14
Gender (female)	12 (24%)
Body surface area (m^2^/kg)	2.0 ± 0.2
Time after transplant (years)	4.7 ± 3.3
NYHA class	1.4 ± 0.6
Histological rejection	24(48%)
Hypertension	29 (58%)
Diabetes	20 (40%)

Data are mean ± SD.

**Table 2:** Echocardigraphic measurements

Variables	
Left ventricular ejection fraction (%)	67 ± 7
Left ventricular end diastolic volume (ml)	68 ± 19
Indexed Left ventricular end diastolic volume (ml/m^2^)	34 ± 9
Left atrial volume (ml)	83.5 ± 23.7
Indexed-left atrial volume (ml/m^2^)	40.6 ± 11.5
Change in left atrial volume (ml/year)	3.9 ± 17.6
Indexed-change in left atrial volume (ml/year/m^2^)	1.9 ± 8.5
Mitral inflow peak diastolic E velocity (cm/sec)	85.0 ± 23.1
Mitral inflow peak diastolic A velocity (cm/sec)	41.3 ± 13.5
Mitral valve inflow E/A	2.3 ± 1.1
Peak diastolic E velocity of lateral mitral annulus	13.8 ± 3.7
E/E'	6.8 ± 3.3

E = diastolic early filling. A = diastolic atrial contraction. E/A = ratio of peak E velocity to A velocity of mitral inflow. E/E' = ratio of peak E mitral inflow velocity of peak E velocity of lateral mitral annulus. Data are mean ± SD. n = 50 except change in left atrial volume (n = 40).

### Relationship between LAV and ΔLAV and exercise test characteristics

**Figure 2:**
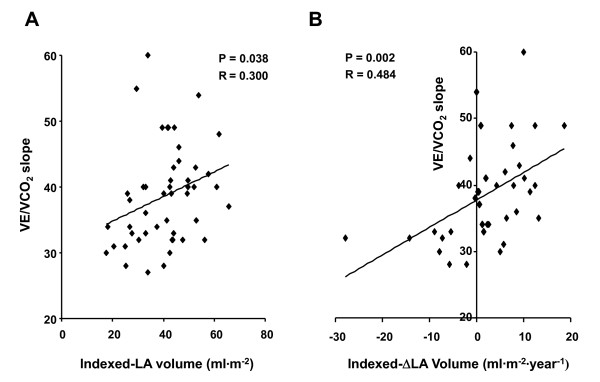
**Relationship between left atrial volume and ventilatory efficiency**. The linear correlation between left atrial (LA) volume in panel A or yearly change in LA volume (ΔLA) volume with ventilatory efficiency (VE/VCO_2 _slope) in panel B is shown. The correlation was analyzed with the Pearson product moment correlation.

All exercise parameters were significantly augmented during exercise in these patients (Table 3), with the exception of diastolic blood pressure. Neither the VE/VCO_2 _slope (r = -0.012, p = 0.934) nor peak VO_2 _(r = 0.010, p = 0.487) correlated with duration post HT, indicating that changes in CPX parameters are not time dependent in this group. However, these findings did not preclude a time dependence of CPX parameters at an individual level. A significant correlation was noted between both absolute LAV and ΔLAV and the VE/VCO_2 _slope (Figure 2). When the patients were classified according to positive and negative values of indexed ΔLAV, those with positive ΔLAV (increasing LA size over one year) showed a significantly higher VE/VCO_2 _slope as compared with those with negative values (40.2 ± 6.5 vs. 33.6 ± 5.0, p = 0.003). Left atrial volume correlated with peak VO_2 _(r = -0.328, p = 0.020) while the correlation with ΔLAV was not significant (r = 0.079, p = 0.616 for those not indexed, r = 0.006, p = 0.971 for those indexed).

**Table 3:** Exercise measurements

Variables	N = 50
Baseline heat rate (bpm)	89 ± 14
Baseline systolic blood pressure (mmHg)	125 ± 18
Baseline diastolic blood pressure (mmHg)	78 ± 11
Baseline pressure rate product (bpm·mmHg·10^3^)	1.09 ± 0.20
Peak exercise heart rate (bpm)	134 ± 18*
Peak exercise systolic blood pressure (mmHg)	161 ± 27*
Peak exercise diastolic blood pressure (mmHg)	81 ± 14
Peak exercise pressure rate product (bpm·mmHg·10^3^)	2.16 ± 0.49*
Peak respiratory exchange ratio	1.13 ± 0.09
Peak exercise oxygen consumption (ml O_2·_min^-1·^kg^-1^)	17.7 ± 6.0
Peak exercise VE/VCO_2 _slope	38.7 ± 7.5

Data are mean ± SD. *P < 0.01 vs. baseline measurements. bpm denotes beat per minute. The comparison of measurements between at baseline and at peak exercise was performed with a paired Student t-test.

## Discussion

The results of the present study demonstrate that in this cohort of HT patients, abnormalities in the exercise response is modest but significantly correlated with both the magnitude of baseline post-HT LAV, as well as positive change in LAV over one year's time (ΔLAV), as reflected by their relationship with ventilatory efficiency (i.e. the VE/VCO_2 _slope). Thus, the association of increased LAV with an abnormal exercise response presents a possibility that left atrial remodeling may be a surrogate for factors limiting the physiologic response to exertion in HT recipients.

It has been proposed that increasing LAV reflects chronic changes in left ventricular diastolic function [26]; therefore, left ventricular diastolic dysfunction may play a role in the pathophysiologic mechanisms that reduce exercise capacity in several different cardiac populations. Although our study population did not show abnormal baseline left ventricular diastolic function parameters with echocardiography, it is possible that this is still a mechanism related to limited exercise capacity with larger LAV, in part because left ventricular diastolic dysfunction frequently may only become evident during exercise while remaining undetected in studies done at rest [27,28]. Only 4 patients (8%) in the current study demonstrated elevated baseline blood pressure; however, 58% of our patients had a history of hypertension. Thus, our study population may be susceptible to exercise-induced left ventricular diastolic dysfunction. In this regard, a future study using exercise echocardiography to assess exercise left ventricular diastolic function in this population could be quite revealing.

The dilatation of LAV might be also in part related to the surgical scar of the left atrial anastomosis. The surgical scar between the native and the donor atrium may impede correct left atrial pump function and therefore, the left atrium may subsequently dilate to increase the reservoir capacity as a compensatory mechanism, which in turn theoretically would maintain left atrial output in the presence of impaired atrial pump function.

Following HT, an enlarged left atrium is considered to be a typical and clinically insignificant finding during any post-transplant echocardiography. This fact often leads to an under-appreciation of how left atrial enlargement may play a role in transplanted heart function. Thus, increases in left atrium size in HT patients, as well as in other cardiac disease patients [9,13], may be an important surrogate for significant loss of atrial function or worsening of left ventricular diastolic function, and furthermore, such functional deterioration may only appear during exercise. For example, as a possible atrial structure-function mechanism, consider that in an enlarged left atrium with preserved wall compliance but without compensatory augmentation of active atrial contraction - as would be the case after HT - with exercise there may be pooling of intra-atrial venous return; such pooling could lead to a significant restriction of left ventricular preload during the period of increased cardiac demand, and therefore in turn limit the patient's exercise capacity. Thus, improved functional capacity in HT recipients with total orthotopic HT using both bicaval and pulmonary vein anastomosis, as compared to traditional orthotopic HT technique, may be in part related to reduction of left atrial size [29]. This hypothesized mechanism might be investigated by assessing left atrial volume and function and exercise capacity in our HT population using exercise echocardiography. Our study for the first time suggests that both indicators - larger absolute LAV and an increase in LAV following HT - may be early warning signs of declining exercise capacity in this population.

The correlation between ΔLAV and CPX measures of peak aerobic capacity was considerably weaker than the correlation with ventilatory efficiency in the present study. Previous work in patients with non-obstructive hypertrophic cardiomyopathy has also found that the linkage between LAV and ventilatory efficiency was stronger compared to that found between LAV and VO_2 _at peak exercise [9,13]. Other investigations in patients with heart failure rather consistently demonstrate that the relationship between various markers of cardiovascular pathophysiology (β-type natriuretic peptide, pulmonary vascular pressures, pulmonary diffusion capacity, etc) and ventilatory efficiency is stronger than the correlation found with peak VO_2 _[30]. A primary reason for the present and past correlation difference may be the reliance that a true peak VO_2 _response has on maximal subject effort, a prerequisite that is not required for attainment of a physiologically valid measure of ventilatory efficiency.

The retrospective nature of this study and relatively small sample size are the primary limitations of the present investigation. While the demonstrated correlation of LAV and exercise capacity holds potential clinical significance, the relationships presented in the present study are numerically relatively modest, indicating that additional factors are likely associated with the CPX response in patients undergoing HT or LAV may be a surrogate for factors that affect exercise capacity rather than a primary determinant. To further strengthen our findings, a prospective study addressing these issues in a larger HT cohort is required. It is also possible that new echocardographic parameters obtained from emerging technology, such as strain/strain rate assessment [31], or more accurate assessment of LAV with other imaging modality may better correlate with exercise performance.

## Conclusion

In conclusion, our study shows that increasing LAV is significantly associated with the limited exercise capacity of HT recipients. Further investigation to evaluate the relationship between LAV and exercise capacity in the HT population is therefore warranted.

## Competing interests

The authors declare that they have no competing interests.

## Authors' contributions

MAW carried out collection of data, data analysis, and editing the manuscript. MY participated in study design, collection of data, and editing the manuscript. SJK participated in collection of data, editing the manuscript. RA participated in study design and editing the manuscript. JY participated in study design and editing the manuscript. NT participated in study design and editing the manuscript. SHD participated in study design and editing the manuscript. YS carried out study design and coordination, collection of data, data analysis, and drafting the manuscript. All authors read and approved the final manuscript.
